# Knowledge, Attitude, and Practice Regarding Breast Cancer Early Detection Among Women in a Mountainous Area in Northern Vietnam

**DOI:** 10.1177/1073274819863777

**Published:** 2019-07-23

**Authors:** Do Thi Thanh Toan, Dinh Thai Son, Le Xuan Hung, Luu Ngoc Minh, Dinh Le Mai, Luu Ngoc Hoat

**Affiliations:** 1 Institute of Preventive Medicine and Public Health, Hanoi Medical University, Hanoi, Vietnam; 2 National Cancer Hospital, Hanoi, Vietnam

**Keywords:** breast cancer early detection, ethnic minority women, Vietnam

## Abstract

Breast cancer is the most common cancer in women all over the world, also in Vietnam. In recent years, the incidence of breast cancer has been increasing in Vietnam, and most cases are diagnosed at late stages, making treatment more difficult. More and better early detection could help more women to survive. The aim of this study was to identify the current knowledge, attitude and practice about early detection of breast cancer as well as potential predictors of breast cancer screening among women aged 20 to 49 year in a mountainous commune in Thanh Hoa Province, Vietnam, in a largely ethnic Muong population. Women aged 20 to 49 years were selected by systematic random sampling to participate in a cross sectional study in October 2017. They were interviewed with a closed questionnaire about their knowledge of breast cancer, its risk factors, and warning signs. A checklist for performance of breast self-examination was also applied. Three hundred six women agreed to participate in the study. More than half had a low level of knowledge, and were weak in attitude and practice about breast self-examination, clinical breast examination, breast ultrasound, and mamography. Among women who had practiced at least 1 screening method, 17.0% mentioned clinical breast examination, and only 13.8% reported practicing breast self-examination. Factors associated with practice included knowledge about breast cancer early detection (BCED), ethnicity, income, the BCED information approach, and the BCED screening programs approach. The finding of a very low proportion of women in the mountainous setting with good awareness and practice on early detection of breast cancer is important evidence to inform the BCED intervention program developers about where and how to target which information, especially to reach more ethnic minority women.

## Introduction

Breast cancer is the most common cancer in women worldwide. Recent evidence shows that in middle-income developing countries, breast cancer is replacing cervical cancer as the number 1 cause of death from malignant tumors among women.^1^ Almost half of the breast cancer cases detected annually are found in low- and middle-income countries. In 2012, 1.68 million new cases were diagnosed and there were 522 000 deaths from breast cancer around the world.^2^

In Vietnam, breast cancer is the leading cancer among women. The age-standardized rate per 100 000 was 17.5 in 2010. It is the third leading cause of death among women.^3^ In 2012, 11 060 cases of female breast cancer were diagnosed, with 64.7% of the cases occurring in women younger than 50 years.^4^ A large number of breast cancers in Vietnam are detected only at a later stage of development, making the treatment more difficult and much more expensive.^5^

Although it is not possible to prevent breast cancer, the risk of severe disease and death can be reduced through specific prevention activities, among which early detection is the most important.^6^ The most appropriate measures for early detection of breast cancer are breast self-exams, clinical breast examinations, breast ultrasounds, and mammograms. To develop appropriate and effective intervention programs, it is necessary to find out what women already know about these methods and how they are currently applied. The situation in the mountainous regions is special in that most of the inhabitants are of ethnic minorities, with different languages and levels of education than in the lowlands and cities.^7^ In these locations, interventions may need to targeted specifically to the knowledge, attitudes, and practices of the local population.

The promotion of breast cancer early detection (BCED) includes enhancing women’s knowledge about the methods used and their attitudes to practicing them. There is limited information about this topic among ethnic minority women. The aim of our research was to identify the current BCED knowledge, attitude and practice, and potential predictors of breast cancer screening practices among women in an area where 71% of the population are ethnic Muong, one of the ethnic minorities in Vietnam.

## Methods

### Study Setting, Sample Size, and Sampling Method

A cross-sectional survey was conducted in October 2017 in a mountainous commune in Thanh Hoa, a coastal province in Northern Vietnam, where 71% of the population belongs to Muong ethnic minority. In this area, breast cancer screening activities are not yet widely available. Women of reproductive age were selected, from 20 to 49 years (because 20 is the recommendation age to start with clinical examination for breast cancer). Those who had been living for at least 1 year in the commune were selected by systematic random sampling from the list of women collected from the Commune Health Center. Finally, 306 of the 310 women approached agreed to participate. They were then invited to be interviewed and answered a prepared questionnaire (4 women refused either because of poor timing or from reluctance to interact with strangers from another ethnic group).

### Measures and Instruments

The questionnaire was divided into 4 sections; the first covered demographic characteristics and sociocultural status of the respondents; the second included questions related to knowledge of breast cancer, its risk factors, and warning signs. The last 2 sections covered the attitude and practices of the interviewees on early detection of breast cancer. Most of the questions were closed, with “yes” or “no” as possible answers. In the case of the survey on practice, the women were requested to demonstrate how they performed breast self-examination; their performance was assessed by the observer/interviewer, using a checklist. A pilot study was conducted among 30 women to test the questionnaire before running the large study.

### Data Analysis

The data were checked for error and, finally, were analyzed using STATA 14.0 software. Demographic characteristics are presented as frequencies and cross-tabulations. Univariate analysis was applied to calculate the odds ratio (OR) with 95% confidence intervals (95% CIs) for dependent binary variable (BCED practice) and each independent variable (age, ethnicity, marital status, number of children, personal income, knowledge about BCED, and BCED information approach). All variables found to have a statistically significant association (*P* value < .1) with breast cancer screening in the univariate analysis were included in the multivariable stepwise logistic regression models. Adjusted OR with 95% CIs was reported in the final model. The final model was the best-fit model with the lowest Akaike’s Information Criterion. Log-likelihood is a measure of model fit.

## Results

### Sociodemographic Characteristics

Table 1 shows that approximately half of the participants were aged between 30 and 39 years (46.1%); the mean age was 32 years and the standard deviation was 6.6. The majority of respondents was ethnic Muong (67.3%). Almost 50% of the interviewed women had a high school educational level. The main occupations of the participants were farmers (38.2%) or workers (36.3%).

**Table 1.** Characteristics of Respondents.

Variables	N (306)	%
Age (years)	Mean (SD): 32 (6.6) Min: 20, Max: 49	
Age group		
20-29	115	37.6
30-39	141	46.1
40-49	50	16.3
Ethnic		
Kinh	90	29.4
Muong	206	67.3
Other	10	3.3
Education		
Illiteracy	3	1.0
Primary	16	5.2
Secondary	74	24.2
High school	150	49.0
Higher	60	20.6
Occupation		
Farmer	117	38.2
Worker	111	36.3
Sale/service worker	29	9.5
Government staff	41	13.4
Others	8	2.6
Average income		
<1.5 million Vietnam Dong	168	56.2
≥1.5 million Vietnam Dong	131	43.8

Abbreviation: SD, standard deviation.

Most of the study participants were married (95.5%) and had 2 (52.0%) children. Nearly one-third (32.0%) of the women had a history of breast conditions, such as mammary gland rash, breast abscess, or breast cancer. A small minority (9.8%) reported a family history of breast cancer, while 38.6% of the women had friends, neighbors, or colleagues with a history of breast cancer.

### Knowledge on BCED

Among the study population, 247 of 306 patients had knowledge about breast cancer, but more than half demonstrated a low level of knowledge about early detection of breast cancer (155 of 247, or 62.8%).

**Figure 1.**
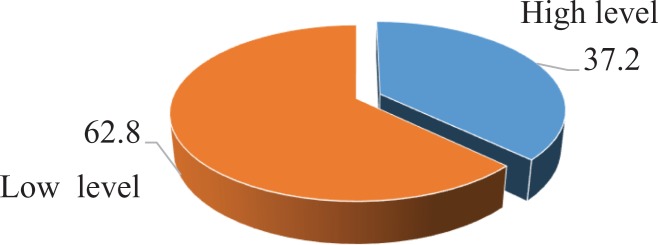
Knowledge level on breast cancer early detection among women aged 20 to 49 years.

Approximately half of the participants (145 of 247 or 58.7%) perceived breast self-examination as a part of breast cancer prevention, but among them, only 32.4% (47 of 145) knew that the frequency for breast self-examination should be once a month and only 19.3% (28 of 145) knew it should begin at age 20 (Figure 1).

More than half (142 of 247 or 57.5%) of the women displayed knowledge related to clinical breast examinations, reporting that women should have a clinical breast examination every 1 to 3 years starting at age 20 and every year starting at age 40. Breast ultrasound was less familiar; more than half of the respondents (144 of 247 or 58.5%) knew nothing about it. Among those who had heard of breast ultrasound, most (81 of 103, 78.2%) thought it was used for treatment when a problem has already been identified, not for early detection. Again around half (115 of 247, 46.6%) of the women knew that x-ray mammography is used as a main method in diagnosis and treatment of breast cancer, but many of them (46 of 115, 40.1%) had very little knowledge on mammography.

### Attitudes to Early Detection

**Figure 2.**
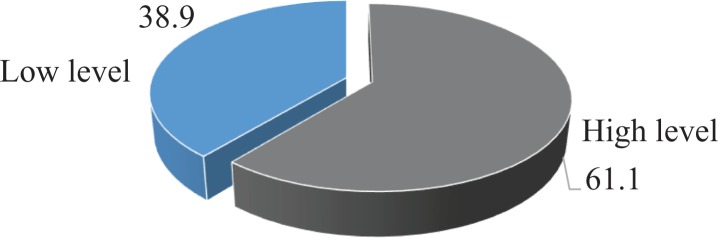
Attitude level on breast cancer early detection among women aged 20 to 49 years.

We then examined participants’ attitudes about early detections of breast cancer. We asked them whether they were likely to want to seek early detection and whether BCED was useful or not. More than half (187 of 306, 61.1%) of participants had a positive attitude to BCED and expressed a preference for mammography and breast ultrasound (Figure 2).

### Breast Cancer Early Detection Practice

**Figure 3.**
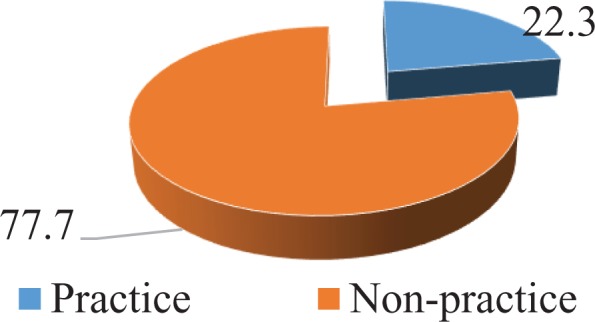
Practice on breast cancer early detection among women aged 20 to 49 years.

**Figure 4.**
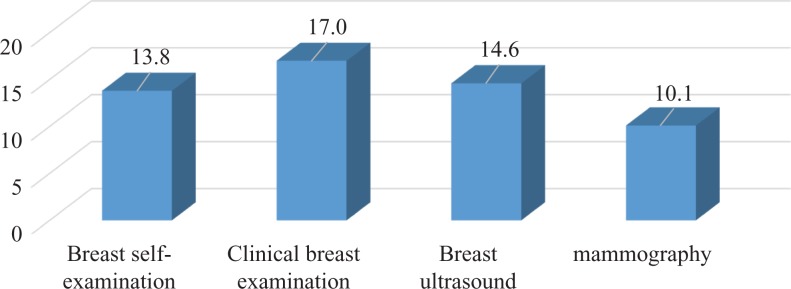
Distribution of breast cancer screening methods among women aged 20 to 49 years.

Among the study population, 247 of 306 women knew about breast cancer. However, most of them had no practice for early detection (192 of 247, 77.7%). Only a small number of participants reported already having done breast self-examination (34 of 247, 13.8%) or having had a clinical breast examination (42 of 247, 17%), breast ultrasound (36 of 247, 14.6%), or mammography (25 of 247, 10.1%). Figures 3 and 4 cannot be added up to make a total of the number of women because there were a few people who chose 2 or more selections (Table 2).

**Table 2.** Factors Associated With Practice on Breast Cancer Early Detection Among Women Aged 20 to 49 Years.^a^

Variable/Predictor	Crude OR (95% CI)	Adj-OR^b^ (95% CI)
Ethnic		
Kinh	1	1
Muong	0.66 (0.38-1.16)	0.33^c^ (0.15-0.69)
Others	1.78 (0.47-6.69)	1.07 (0.12-9.57)
Personal income		
<1.5 million Vietnam Dong	1	1
≥1.5 million Vietnam Dong	0.33^c^ (0.17-0.65)	0.32^d^ (0.12-0.84)
Access to BCED information		
Yes	1	1
No	0.11^c^ (0.04-0.31)	0.23^d^ (0.07-0.77)
Access to BCED screening programs		
Yes	1	1
No	0.03^c^ (0.01-0.14)	0.06^c^ (0.01-0.35)
Knowledge on BCED		
High level	5.12^c^ (2.69-9.75)	3.48^c^ (1.52-7.97)
Low level	1	1

Abbreviations: Adj-OR, adjusted OR; BCED, breast cancer early detection; CI, confidence interval; OR, odds ratio.

^a^AIC = 194.38; constant of model β_0_ = 16.2; pseudo-*R* ^2^ of logistic regression model = 0.237.

^b^Adjusted for education, occupation, and attitude toward BCED.

^c^ *P*< .01.

^d^ *P* < .05.

### Predictors of Breast Cancer Screening

Breast cancer screening practice was found to depend on knowledge about BCED, ethnicity, personal income, BCED information approach, and the BCED screening program approach. The women who had a low level of knowledge on BCED were 0.29 times less likely to practice BCED (OR: 0.29, 95% CI: 0.12-0.66). Women who were Muong ethnic minority had poorer practices on BCED compared to the ethnic majority Kinh women (OR: 0.31, 95% CI: 0.14-0.71). Those women with personal monthly income above 1.5 million VN Dong were less likely to practice (OR: 0.32, 95% CI: 0.12-0.84). Related to accessing information on BCED and the BCED screening program, those who had less access also had less practice (OR: 0.23, 95% CI: 0.07-0.77; OR: 0.06, 95% CI: 0.01-0.35, respectively).

## Discussion

Breast cancer is currently the most common cancer among Vietnamese women.^4^ The age group most commonly affected in Vietnam is from 45 to 55 years. Most women are diagnosed at later stages of the disease; early detection is still not well developed in the country. We recruited women from 20 to 49 years, living in a remote area; our study population had a mean age of 32 and most were of the Muong ethnic minority. More than half of the women had a low level of knowledge, attitudes, and practice with regard to the key screening measures: breast self-examination, clinical breast examination, breast ultrasound, and mammography. Similar findings arose in other Asian countries such as India and Malaysia.^8-10^ A study in India among university students, with a high educational level, revealed that fewer than half of were aware of the need to do breast self-examination.^8^ Since just over 10% of the women in our study reported having used self-examination, it is perhaps not surprising that their performance was also not very good, when they were asked to demonstrate how to do it. Another study among women aged 40 to 74 years attending a primary care clinic in Malaysia found that 71% of them had little knowledge about risk factors for breast cancer and about early screening.^10^

Several previous studies have reported that a large proportion of patients with breast cancer in Vietnam and Asia countries are diagnosed with advanced stages of the disease; up to 25% have distant metastases at initial presentation.^11,12^ Following the World Health Organization guideline for breast cancer screening programs, a number of Asian countries have introduced nationwide screening policies. A national program has recently been established in Vietnam as well, but its screening activities are not yet widely available. The major barriers to screening in our study were the lack of knowledge about the benefits of early detection and lack of access to BCED information and BCED screening program, and these women were most frequently found among women of the Muong ethnic minority. As revealed from a study conducted in other mountainous area, Muong women were influenced by their ethnic traditions, which limited the practice of breast cancer screening methods.^7^

Apart from knowledge of BCED, the performance of breast cancer screening also depends on women’s attitudes and cultural habits. In some countries, such as India, local culture may inhibit women from breast self-examination.^8^ In Vietnam, culture also affected the Muong women interviewed; they felt embarrassed to discuss breast self-examination, so their poor performance while observed may also have been influenced by shyness. The national program is not yet available to these women, which could also explain their low knowledge and lack of interest in screening and self-examination. Developed countries have reported a higher rate of breast cancer screening than most developing countries, possibly because women in developed countries have more opportunities to learn from educational programs on breast cancer and can access supportive services.^13^ Under the current national program, screening activities are mainly carried out in the large cities in Vietnam,^12^ so for women living in mountainous commune, breast self-examination is currently the best recommendation. They would, however, also have to be informed about where to go and what to do if they do find a suspicious lump upon self-examination. Improving knowledge, attitude, and practice for women about BCED, especially breast self-examination, should be considered from an early adult age, because it can lead to early intervention and diagnosis of breast cancer, which in turn leads to increased survival.

Several limitations exist in our study. First, it was done in only one commune and the sample was not completely random, so the findings may not be generalized to all women in the mountainous communes of Thanh Hoa Province or other mountainous areas. However, nearly all of the suitable women in that commune were willing to participate in the study, and this commune is typical for its area, so we do expect that a larger study would find similar results. Second, due to limited time and difficult access to and communication with the ethnic minority women, we were not able to conduct a qualitative study to obtain a deeper understanding of the factors associated with knowledge, attitude, and practice of women on BSED. Such a study would increase our understanding and guide interventions to improve early screening for breast cancer.

## Conclusion

We observed that the majority of women in the mountainous commune of Thanh Hoa Province demonstrated a poor knowledge, attitude, and practice of breast cancer screening methods. An effective breast self-examination would most likely be beneficial to women in such a mountainous area as it is elsewhere. Additional long-term efforts are needed to increase the coverage of the national breast cancer program to reduce loss of life from breast cancer by increasing detection at early stages that can be more successfully treated. Special attention will be needed to ensure that women in remote areas, especially those with different ethnic backgrounds, have sufficient information and encouragement to join in practicing early detection.
